# Translation Stress Positively Regulates MscL-Dependent Excretion of Cytoplasmic Proteins

**DOI:** 10.1128/mBio.02118-17

**Published:** 2018-01-30

**Authors:** Rosa Morra, Francesco Del Carratore, Howbeer Muhamadali, Luminita Gabriela Horga, Samantha Halliwell, Royston Goodacre, Rainer Breitling, Neil Dixon

**Affiliations:** a Manchester Institute of Biotechnology, School of Chemistry, University of Manchester, Manchester, United Kingdom; Korea Advanced Institute of Science and Technology

**Keywords:** ArfA, MscL, osmotic stress, protein excretion, translation stress

## Abstract

The apparent mislocalization or excretion of cytoplasmic proteins is a commonly observed phenomenon in both bacteria and eukaryotes. However, reports on the mechanistic basis and the cellular function of this so-called “nonclassical protein secretion” are limited. Here we report that protein overexpression in recombinant cells and antibiotic-induced translation stress in wild-type *Escherichia coli* cells both lead to excretion of cytoplasmic protein (ECP). Condition-specific metabolomic and proteomic analyses, combined with genetic knockouts, indicate a role for both the large mechanosensitive channel (MscL) and the alternative ribosome rescue factor A (ArfA) in ECP. Collectively, the findings indicate that MscL-dependent protein excretion is positively regulated in response to both osmotic stress and *arfA*-mediated translational stress.

## IMPORTANCE

Protein translocation is an essential feature of cellular organisms. Bacteria, like all single-cell organisms, interact with their environment by translocation of proteins across their cell membranes via dedicated secretion pathways. Proteins destined for secretion are directed toward the secretion pathways by the presence of specific signal peptides. This study demonstrates that under conditions of both osmotic stress and translation stress, *E. coli* cells undergo an excretion phenomenon whereby signal peptide-less proteins are translocated across both the inner and outer cell membranes into the extracellular environment. Confirming the presence of alternative translocation/excretion pathways and understanding their function and regulation are thus important for fundamental microbiology and biotechnology.

## INTRODUCTION

Bacteria use a variety of secretion systems to translocate proteins across their cell membrane(s). These systems are involved in a range of essential cellular functions facilitating bacterial virulence, cell attachment, and nutrient scavenging. In Gram-negative bacteria, six secretion pathways (T1SS [type 1 secretion system] to T6SS) have been identified. The T1SS, T3SS, T4SS, and T6SS pathways all translocate proteins in a single step from the cytoplasm to the extracellular environment. In contrast, the T2SS and T5SS pathways secrete proteins in a two-step process in which proteins are first translocated across the inner membrane (IM) by either the general secretion (SecYEG) pathway or the twin arginine (Tat) pathway and then across the outer membrane (OM) in a T2SS/T5SS-dependent manner (1). In addition, a number of periplasmic *Escherichia coli* proteins have been discovered to be translocated to the extracellular medium without being associated with one of the classical OM secretion pathways under standard laboratory cultivation conditions (2, 3). A considerable body of evidence suggests that bacterial cells are also capable of releasing proteins to the extracellular environment in the absence of a signal sequence, and these observations are collectively referred to as nonclassical secretion (4, 5), excretion of cytoplasmic protein (6, 7), or moonlighting proteins (8). Nonclassical secretion has primarily been observed in Gram-positive bacteria; however, analysis of the extracellular proteome of laboratory *E. coli* strains has identified a significant number of cytoplasmic proteins (9). To date, no generalized mechanism has been elucidated and the topic is controversial, in particular as a number of cytoplasmic proteins characterized as being released via nonclassical secretion have subsequently been found to be released by OM vesicles (OMVs) (5, 10, 11).

In this study, we observed that during *E. coli* cell culture, signal peptide-less recombinant protein is localized in the medium at a titer of 0.7 g/liter and up to 80% purity in a lysis-independent manner. Analysis of growth medium osmolality and the metabolic footprint indicates that a hypo-osmotic stress is linked to the excretion phenomenon. We observed that proteins are excreted across the IM into the periplasmic space in a large-conductance mechanosensitive channel (MscL)-dependent manner. Condition-specific proteome analysis additionally found translation stress response signatures to be highly associated with this excretion, which was validated by genetic knockout. Collectively, the findings suggest a direct linkage of osmotic stress, the alternative ribosome rescue factor A (ArfA)-mediated response to translational stress, and MscL-dependent excretion. Finally, the findings were also validated in a wild-type (nonrecombinant) background, confirming the physiological relevance of the excretion phenomenon.

## RESULTS

### Recombinant proteins observed in *E. coli* culture medium during late exponential growth phase.

**FIG 1:**
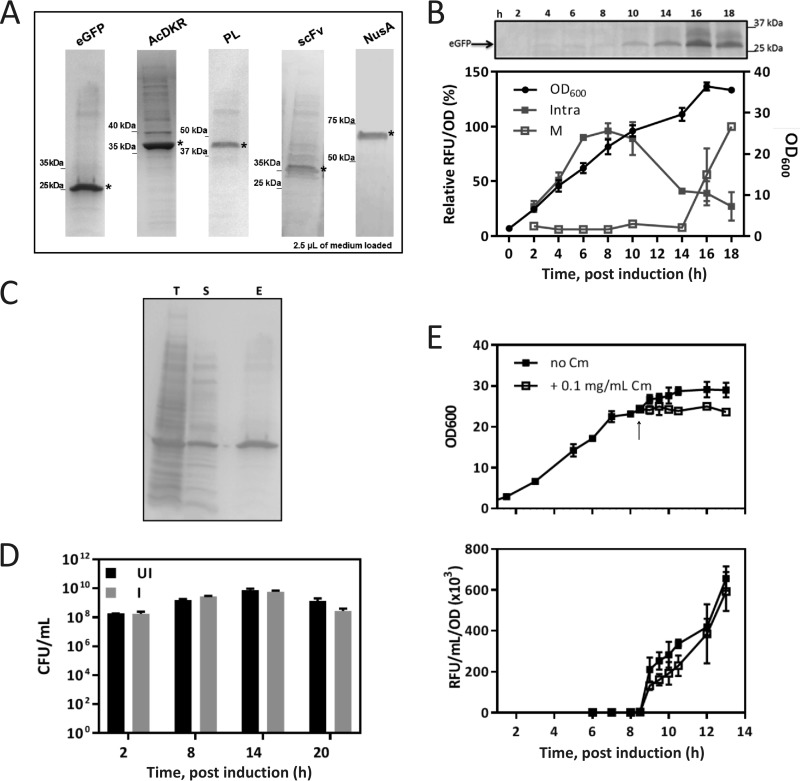
Analysis of extracellular protein profile and viability of *E. coli* during recombinant protein expression. (A) Extracellular protein profile of *E. coli* BL21(DE3)pET-GOI during transition/early stationary phase. The recombinant protein is indicated by an asterisk. (B) Intracellular (Intra) and medium (M) localizations of eGFP and cell growth (OD_600_) over time monitored by SDS-PAGE (M) and RFU normalized to cell density (RFU/OD_600_). (C) SDS-PAGE of the total intracellular fraction (T), the soluble intracellular fraction from sonicated cells (S), and the extracellular fraction (E) at 20 h postinduction. (D) CFU assays in the absence (UI) and presence of induction (I, 250 μM IPTG) over time. (E) Cell growth (top) and extracellular eGFP localization (bottom) monitored after addition of Cm at the time indicated by the arrow. In panels B, D, and E, data are the mean of at least three biological replicates ± the standard deviation.

In the expression of signal peptide-less recombinant proteins in an *E. coli* BL21(DE3) host, enhanced green fluorescent protein (eGFP; 26 kDa), 3-hydroxybutyryl-coenzyme A dehydrogenase (AcDKR; 30 kDa), pectate lyase (PL; 44 kDa), IgG short-chain antibody fragment scFv13R4 (28 kDa), and transcription termination/antitermination protein NusA (58 kDa) all unexpectedly led to extracellular accumulation 10 to 12 h after induction (Fig. 1A and B). Protein quantification showed a yield of ~0.7 g/liter (90 mg/g of dry cell weight) and a relative purity of 60 to 80% at 20 to 24 h postinduction (see Fig. S1A in the supplemental material). Importantly, all of the recombinant proteins tested retained their corresponding biological activity (Fig. S1B and C) and localized in the extracellular medium with relatively high purity.

10.1128/mBio.02118-17.1 FIG S1  Characterization of excreted proteins. (A) Extracellular protein profile of *E. coli* BL21(DE3) host expressing eGFP (GI) assessed by SDS-PAGE stained with Sypro Red and the percentage of eGFP protein calculated by densitometry. (B) Binding of the anti-β-Gal short-chain antibody fragment (scFv13R4) to β-Gal isolated from the periplasmic fraction of the *E. coli* expression host BL21(DE3). (C) AcDKR activity, assessed by ethyl 4-chloroacetoacetate reduction, isolated from the extracellular fraction of the *E. coli* BL21(DE3) expression host and detected spectrophotometrically via NADH oxidation (340 nm). (D) SDS-PAGE densitometry analysis of the normalized total intracellular, soluble intracellular, and extracellular protein profile at 20 h postinduction. (E) SDS-PAGE analysis of extracellular eGFP localization in the absence and presence of Cm. In panels A and B, data are the mean value ± the standard deviation of at least three biological replicates. Download FIG S1, PDF file, 0.3 MB. Copyright © 2018 Morra et al. 2018 Morra et al. This content is distributed under the terms of the Creative Commons Attribution 4.0 International license.

### Extracellular localization of recombinant protein is not due to cell lysis.

General cell lysis was assessed by examination of the protein profiles of different cellular fractions at 20 h postinduction (Fig. 1C). The protein profile of the extracellular sample is significantly different from that of total and soluble intracellular samples. This suggests that lysis is not occurring, as this would result in the extracellular profile being essentially identical to the soluble intracellular profile (Fig. S1D). In a CFU assay, no significant difference in viability was observed between the uninduced and induced conditions for the first 14 h postinduction, further indicating that the release of recombinant protein is not due to cell death (Fig. 1D). In accordance with previous reports, variation in viability among the lag, exponential, and stationary phases was observed (12).

### Extracellular localization of recombinant protein occurs from an existing cellular pool.

To explore whether the lack of an observable effect on the cell growth profile and cell viability are being masked by cell growth and lysis occurring at similar rates, we performed cultivation in the presence of chloramphenicol (Cm). Cm was added (8.5 h), after which cell density remained constant for 4 h, whereas slow growth continued under the control condition (no Cm) (Fig. 1E, top). Under both conditions, extracellular localization of eGFP was observed with no significant difference in the detected signal (Fig. 1E, bottom). This was further confirmed by SDS-PAGE analysis of the extracellular fraction where increasing eGFP protein is observed following Cm addition (Fig. S1E). As no new cell growth or protein synthesis can occur in the presence of Cm, this result indicates that the source of extracellularly localized recombinant protein is an existing pool of cells and already translated protein rather than lysis-dependent release.

### Cells undergoing extracellular release of recombinant protein display a compromised membrane phenotype.

**FIG 2:**
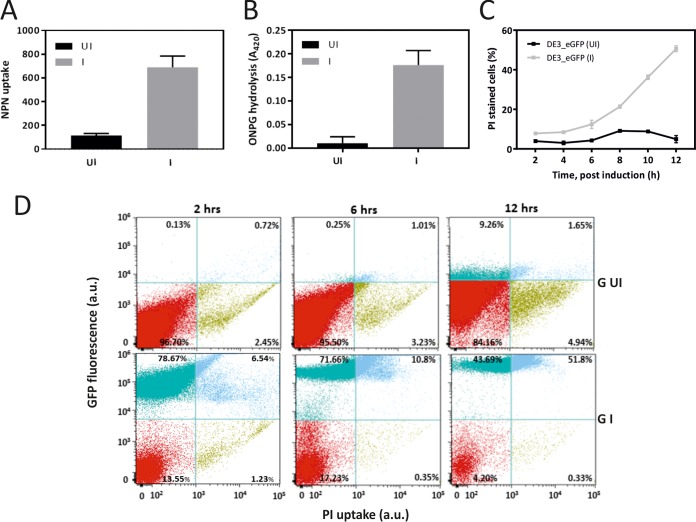
Permeability of the IM and OM of *E. coli* BL21(DE3)pET-GOI during growth. (A) OM permeability (NPN uptake) analysis in the absence (black) and presence (gray) of AcDKR expression. (B) IM permeability (ONPG hydrolysis) analysis in the absence (dark) and presence (gray) of eGFP expression. (C) Cell permeability (PI uptake) analysis over time in the absence (black) and presence (gray) of eGFP expression. (D) FACS density plots of eGFP expression and PI uptake of cells expressing (GI, bottom) or not expressing (G UI, top) eGFP over time. UI and I, no addition and addition of 250 µM IPTG, respectively. Data represent mean values ± the standard errors from independent biological experiments. a.u., arbitrary units.

To investigate cell membrane integrity during cultivation, the permeability of the OM was assessed in a fluorescence-based *N*-phenyl-1-naphthylamine (NPN) uptake assay (13). Because of the incompatibility with eGFP, the assay was performed with cells expressing AcDKR; a 6-fold increase in OM permeability was observed in cells expressing the recombinant protein (Fig. 2A). The permeability of the IM was assessed by monitoring the activity of the endogenous cytoplasmic β-galactosidase (β-Gal) in cells expressing eGFP (Fig. 2B). Minimal activity was observed in the uninduced cells, whereas significantly greater activity (20-fold, *P* = 0.01) was detected in induced cells expressing eGFP. Taken together, both the OM and IM results indicate that the recombinant protein expression increases membrane permeability, suggesting that this may be linked to extracellular localization of the recombinant protein.

The membrane permeability of individual *E. coli* cells was further investigated by measuring the uptake of the fluorescent dye propidium iodide (PI) by flow cytometry (Fig. 2C). Although PI staining of cells is commonly referred to as a viability assay, a number of studies of microbial hosts have shown that membranes are permeable to PI under stressed conditions (14) and that PI-permeable membranes can be repaired (15). Additional studies of *E. coli* have shown that cells become PI stained following hypo-osmotic shock with no significant loss of viability (16). During the exponential and transition growth phases (2 to 12 h), greater membrane permeability (up to 4-fold) was observed in cells that were expressing recombinant protein (Fig. 2C), consistent with the earlier OM and IM permeability analysis (Fig. 2A and B). To assess whether the observed membrane permeability correlates with recombinant protein expression levels, we imaged the colocalization of PI (staining) and eGFP (expression). The majority of induced cells initially (2 h) demonstrated high eGFP expression accompanied by a low level of PI staining (>75%) (Fig. 2D). The level of PI staining gradually increased over time to a maximum at 12 h, at which point >50% of the cell population showed both a high level of PI staining and a high eGFP signal level. In contrast, cultivation in the absence of the inducer demonstrated a low level of PI staining and, as expected, a minimal eGFP signal (Fig. 2D). In summary, these results indicate that extracellular localization is linked to high expression levels rather than the cultivation conditions.

### Signal peptide-less protein localizes in the periplasmic space before leading to extracellular localization.

**FIG 3:**
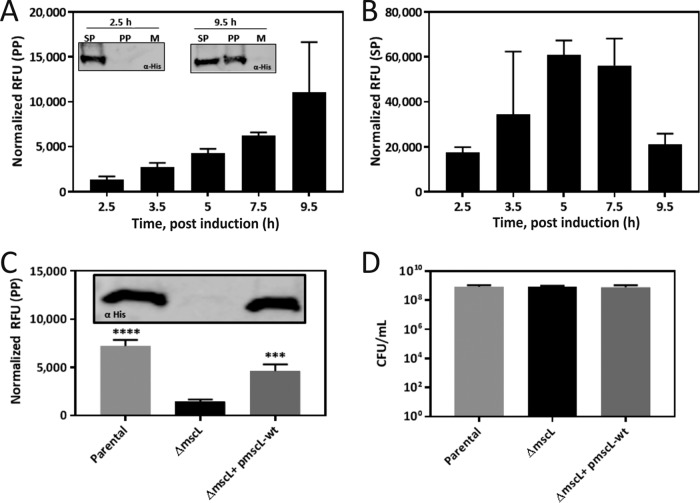
Analysis of recombinant protein localization. (A) Localization of eGFP in the periplasm (PP) over time in BL21(DE3) and representative Western blot analyses (insets). (B) Localization of eGFP in the spheroplast (SP) over time in BL21(DE3). (C) Localization of eGFP in the periplasm of parental, MscL-deficient (Δ*mscL*), and restored (Δ*mscL*/p*mscL*-wt) *E. coli* K-12 cells at 16 h postinduction and representative Western blot analysis (inset). (D) CFU assay of parental, MscL-deficient (Δ*mscL*), and restored *E. coli* K-12 cells. eGFP fluorescence is shown as RFU normalized to cell density. Data are presented as the mean value ± the standard deviation of at least three biological replicates. *, *P* < 0.05; ***, *P* < 0.001; ****, *P* < 0.0001 (two-way ANOVA followed by Dunnett’s multiple-comparison test).

Next we explored whether the signal peptide-less recombinant protein(s) reaches the periplasmic space prior to being released into the extracellular medium. Surprisingly, we consistently detected recombinant protein in the periplasmic fraction during the exponential (Fig. 3A and B; Fig. S2A) and stationary (Fig. S2B) phases. Recombinant overexpressed proteins NusA and scFv were also found to localize in the periplasmic space (Fig. S2C and D). Potential cross contamination during cellular fractionation was excluded by using antibodies against fraction-specific markers (Fig. S2E). *E. coli* BL21(DE3) is a strain specifically engineered for recombinant protein overexpression and carries deletions of the OmpT and Lon proteases and an insertion of the highly active T7 bacteriophage RNA polymerase (RNAP) (17), which confer enhanced expression capability (18, 19). To explore the strain dependency of the phenomenon, we therefore also assessed the localization in *E. coli* K-12 strain BW25113 of recombinant protein expressed from an *E. coli* RNAP-dependent promoter (P_*BAD*_). Recombinant protein was again detected in the periplasmic space of both NusA (Fig. S6A)- and eGFP (Fig. 3C)-expressing strains, although the localization was greatly reduced (14-fold) in comparison to that in the BL21(DE3) strain (Fig. S2B). In addition, little or no protein was detected in the medium, indicating that the extent of periplasmic localization and extracellular localization is dependent on the expression level.

10.1128/mBio.02118-17.2 FIG S2  Protein localization analysis. (A) Production and localization of eGFP in the periplasm (PP) (top) and spheroplast (SP) (bottom) monitored by Western blot analysis (anti-His) during *E. coli* BL21(DE3) cultivation. (B) Production and localization of eGFP in the PP (top) and SP (bottom) monitored by fluorescence assay during *E. coli* BL21(DE3) cultivation. (C) SDS-PAGE analysis of the normalized PP protein profile of *E. coli* host BL21(DE3)pET-NusA uninduced (UI, 0 μM IPTG) and induced (I, 250 μM IPTG) at 12 h postinduction. (D) Production and localization of scFv, monitored by Western blot analysis (anti-His) from *E. coli* BL21(DE3) cultivation at 14 h postinduction (250 μM IPTG). (E) *E. coli* anti-lactamase and anti-RNAP σ^70^ Western blot analysis of normalized cellular fractions (M, centrifuged growth medium; PP, periplasm; SP, spheroplast) at the time point indicated. (F) SDS-PAGE of proteins packaged into the OMVs from host *E. coli* BL21(DE3)pET44eGFP at 20 h postinduction (I, 250 μM IPTG) and uninduced (UI, 0 μM IPTG) (top panel). Western blot analysis of the eGFP, GroEL, and OmpA proteins performed with protein-specific antibodies (bottom) Lanes: 1, filtered culture medium; 2, filtered medium after centrifugation at 35,000 × *g*; 3S, supernatant collected after ultracentrifugation (200,000 × *g*) of the medium; 3P, pellet (OMVs) after ultracentrifugation of the medium. In panels A and B, the data are presented as percent relative signal intensity (signal intensity at *x* hours versus the highest signal intensity measured) and as the mean value ± the standard deviation of three biological replicates. Download FIG S2, PDF file, 0.4 MB. Copyright © 2018 Morra et al. 2018 Morra et al. This content is distributed under the terms of the Creative Commons Attribution 4.0 International license.

### Extracellular localization of recombinant protein is not linked to the formation of OMVs.

It is known that part of the periplasmic space can be encapsulated inside OMVs and its content can be found in the extracellular environment (20). Previous studies have shown that OMVs can transport both periplasmic proteins such as the β-lactamase (21) and indeed abundant cytoplasmic proteins such as GroEL (11). We therefore isolated OMVs as previously described (22) that showed a protein profile similar to that previously reported (11), in particular, association with both OmpA and GroEL (Fig. S2F). In contrast, eGFP found in the extracellular medium was not associated with purified OMVs, indicating that the protein is not exported into the extracellular environment via the action of OMVs.

### Effect of culture medium on recombinant protein localization.

Since excretion of protein into the periplasm was not observed in growth medium containing sodium chloride (Luria broth [LB] or terrific broth [TB] supplemented with sodium chloride) (Fig. S3A and B), we measured the osmolality of both TB and LB growth media before and after cultivation. TB showed a lower osmolality (346 mOsm) than LB (461 mOsm) preinoculation; further, the osmolality of the spent TB (16 h) decreased to ~250 mOsm. In contrast, the osmolality of the spent LB was maintained (~450 mOsm) most likely because of the nature and relatively high concentration of the sodium chloride osmolyte (present at 170 mM). We thus sought to investigate if cellular stress caused by the change in external osmolality in combination with the recombinant protein expression were leading to the excretion phenomenon.

10.1128/mBio.02118-17.3 FIG S3  Medium-dependent localization of eGFP and metabolomic studies. (A) Production and localization of eGFP from BL21(DE3)pET44eGFP in LB and TB at 18 h postinduction (250 μM IPTG). (B) Periplasmic localization of eGFP from BL21(DE3)pET44eGFP in TB in the absence and presence of 200 mM NaCl at 20 h postinduction (250 μM IPTG). The periplasmic localization of eGFP was also confirmed by Western analysis (top panel). (C and D) PCA (C) and PC-DFA (D) of GC-MS analysis of parental (DE3), pET-NusA (E), and pET-eGFP (G) BL21(DE3) cells in the absence (UI) and presence (I) of IPTG (250 μM). The data shown are the normalized relative eGFP fluorescence (RFU/OD/ml) detected in the spheroplast (SP), periplasm (PP), and medium (M) fractions. For panel A, two biological replicates were analyzed and an error bar is shown. The data are the mean values ± the standard errors of three independent biological experiments. *, *P* < 0.05 (one-way ANOVA followed by Tukey’s *post hoc* test). Download FIG S3, PDF file, 0.2 MB. Copyright © 2018 Morra et al. 2018 Morra et al. This content is distributed under the terms of the Creative Commons Attribution 4.0 International license.

### Metabolic profile and footprint indicate extracellular localization of recombinant protein associated with lower levels of osmoprotectant.

**FIG 4:**
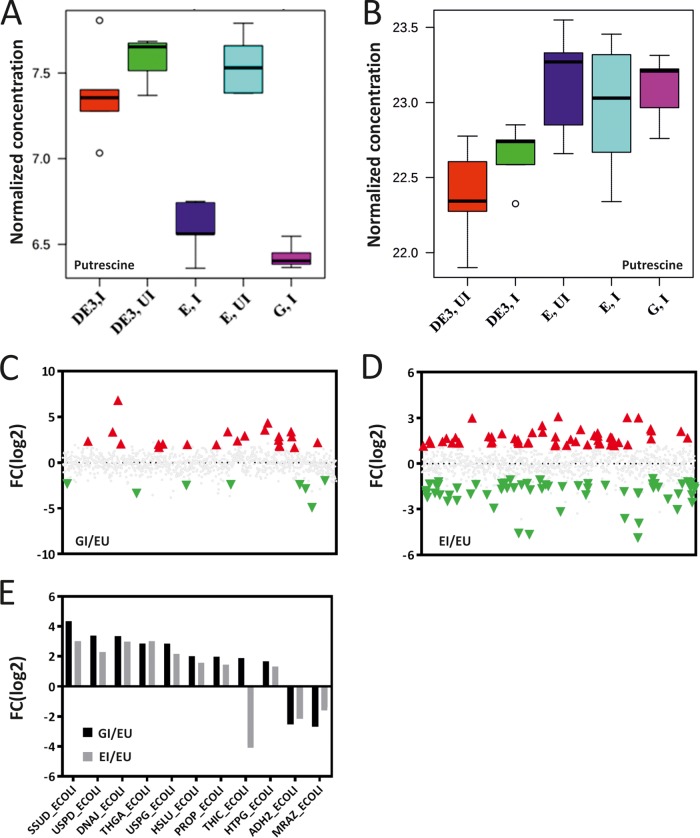
Significant differentially detected metabolites and proteins in cells undergoing excretion. (A and B) Metabolite analysis by GC-MS of putrescine from the medium (A) and cell extract (B) of parental (DE3), pET-NusA (E), and pET-eGFP (G) BL21(DE3) strains in the absence (UI) and presence (I) of IPTG. (C) Induction-specific proteins differentially detected by LC-MS analysis in eGFP-induced (GI) versus NusA-uninduced (EU) strains (red, upregulated; green, downregulated). (D) Induction-specific proteins differentially detected by LC-MS analysis in NusA-induced (EI) versus uninduced (EU) strains. (E) Comparison of proteins differentially detected under both conditions (C and D). In panels A and B, the data are mean values of five biological replicates ± the standard deviation. In panels C to E, data are presented as fold changes [FC(log_2_)] in the normalized mean values for a minimum of four biological replicates of proteins differentially detected with significance (*P* < 0.05) testing performed by the Wilcoxon RS method.

The metabolic effects of recombinant protein expression upon *E. coli* were assessed by using five different induction conditions/strains by gas chromatography-mass spectrometry (GC-MS) of the spent medium (metabolic footprinting) and cell extract (metabolic profiling) (see Materials and Methods). The five specific strains/conditions assessed were an *E. coli* BL21(DE3) strain containing (i) an expression plasmid encoding the inducible green fluorescent protein (eGFP) that has been induced leading to expression of the recombinant eGFP-encoding gene (GI), (ii) an expression plasmid encoding the inducible transcription termination/antitermination protein NusA that has been induced (EI), (iii) an expression plasmid encoding NusA that has not been induced (EU), (iv) no plasmid (DE3,U), or (v) no plasmid in the presence of isopropyl-β-d-thiogalactopyranoside (IPTG) (DE3,I). The BL21(DE3) strain containing eGFP, in the absence of induction, showed significant expression (20% leak) and therefore was not used as a negative control. Figures S4 and S5 highlight metabolites that were statistically significant and allowed differentiation among the five different induction conditions/strains for both the footprint and the profile (Fig. S3C and D). Most interestingly from the footprint, putrescine (*P* < 7 × 10^−4^) was found to be negatively correlated with the induced conditions (EI, GI) that result in extracellular localization of recombinant protein (Fig. 4A). Putrescine is a polyamine and has been found to be associated with the regulation of a number of bacterial stress response mechanisms, including oxidative stress and the hyperosmotic shock response (23, 24). In the metabolic profile, putrescine was found to correlate positively with plasmid presence (Fig. 4B). Taken together, the lower external putrescine levels of the strains producing recombinant protein (EI, GI) and the similar internal putrescine levels of the plasmid-containing strains (EI, EU, GI) seem to indicate that the strains undergoing the excretion phenomenon are experiencing greater hypo-osmotic (less hyperosmotic) conditions.

10.1128/mBio.02118-17.4 FIG S4  Footprint analysis. Normalized peak intensities of the footprint (extracellular medium) metabolites detected by GC-MS in parental (DE3), pET-NusA (E), and pET-eGFP (G) BL21(DE3) cells in the absence (UI) or presence (I, 250 μM) of IPTG. Data are presented as the mean value ± the standard deviation of five biological replicates. *, *P* < 0.05; ***, *P* < 0.001; ****, *P* < 0.0001 (one-way ANOVA followed by Tukey’s *post hoc* test). Download FIG S4, PDF file, 0.5 MB. Copyright © 2018 Morra et al. 2018 Morra et al. This content is distributed under the terms of the Creative Commons Attribution 4.0 International license.

10.1128/mBio.02118-17.5 FIG S5  Metabolic profile analysis. Normalized peak intensities of the metabolic profile (cell extract) detected by GC-MS in parental (DE3), pET-NusA (E), and pET-eGFP (G) BL21(DE3) cells in the absence (UI) or presence (I, 250 μM) of IPTG. Data are presented as the mean value ± the standard deviation of five biological replicates. *, *P* < 0.05; ***, *P* < 0.001; ****, *P* < 0.0001 (one-way ANOVA followed by Tukey’s *post hoc* test). Download FIG S5, PDF file, 0.5 MB. Copyright © 2018 Morra et al. 2018 Morra et al. This content is distributed under the terms of the Creative Commons Attribution 4.0 International license.

### Periplasmic localization requires large-mechanosensitive channel (MscL).

In the literature, there are several reports of cytoplasmic proteins being released into the periplasmic space upon growth medium exchange/osmotic downshock via the mechanosensitive channel MscL (25, 26). However, other studies have suggested that protein release is not dependent upon MscL but is an artifact of the fractionation procedure (27). To explore the possible involvement of MscL in the observed excretion phenomenon, we compared the localization of the recombinant protein expressed from an *E. coli* RNAP-dependent promoter (P_*BAD*_) in *E. coli* K-12 strain BW25113 (parental), an MscL-deficient (Δ*mscL*) mutant strain (28), and a strain with MscL restored (Δ*mscL* mutant plus episomal *mscL*). The results show periplasmic localization of recombinant protein occurring in the parental strain. In contrast, the Δ*mscL* mutant strain displayed a significant decrease in periplasmic localization of eGFP, assessed by fluorescence (5-fold; *P* = 9 × 10^−3^) (Fig. 3C), and a consistent decrease in NusA, assessed by Western blot analysis, was also observed (Fig. S6A). Episomal expression of MscL rescued the parental phenotype, and recombinant protein was again localized in the periplasmic fraction (Fig. 3C). All of the deletion strains assessed demonstrated viability comparable to that of the control strains and literature values (Fig. 3D) (29). We also created a BL21(DE3) *mscL* deletion strain and again observed decreased (14-fold; *P* = 1.3 × 10^−3^) periplasmic localization of eGFP in the absence of MscL (Fig. S6B). This result indicates a direct role for MscL in the excretion of recombinant protein(s) into the periplasm of *E. coli*.

10.1128/mBio.02118-17.6 FIG S6  Periplasm localization of recombinant and native proteins. (A) Western blot analysis (anti-His antibody) of normalized cellular fractions (PP, periplasm; SP, spheroplast) from *E. coli* host K-12 (parental) and MscL knockout (Δ*mscL*) expressing recombinant NusA (pBAD-NusA) at 14 h postinduction (13.3 mM arabinose). (B) Localization of eGFP in the PP of parental and MscL-deficient (ΔMscL) *E. coli* BL21(DE3) cells at 5 h postinduction. (C) Localization of eGFP in the PP of parental and ArfA-deficient (Δ*arfA*) *E. coli* K-12 cells at 16 h postinduction. (D) SDS-PAGE of periplasmic fractions of parental wild-type (nonrecombinant) *E. coli* K-12 (lanes 1 and 3) and a Δ*mscL* mutant (lanes 2 and 4) 10 h after addition of Cm (0.01 mg/ml) (lanes 3 and 4) and no treatment (lanes 1 and 2). All lanes contain fractions obtained from the same number of cells. Lysozyme (labeled) was used for the fractionation procedure. Data are the mean value ± the standard deviation of at least three biological replicates. **, *P* ≤ 0.01 (two-way ANOVA followed by Dunnett’s multiple-comparison test). Download FIG S6, PDF file, 0.2 MB. Copyright © 2018 Morra et al. 2018 Morra et al. This content is distributed under the terms of the Creative Commons Attribution 4.0 International license.

### Proteome analysis.

Having confirmed that the underlying cause of the excretion phenomenon is not cell lysis but that it is dependent upon MscL, we sought to explore whether native regulatory mechanisms are involved; therefore, we investigated the gene expression effects by performing an unlabeled proteomic study (see Materials and Methods). We assessed the same three different expression conditions/strains (EU, EI, GI) used for metabolomic studies, and for each condition, two separate sample fractions (total cell extract and periplasm) were collected and analyzed to allow topological information in addition to relative abundance to be assessed.

### Protein identification in the cell extract.

Principal-component analysis (PCA) of all 15 protein profiles (three conditions, five replicates) was performed to characterize condition clustering and identify any outliers (Fig. S7A and B). In total, 1,075 proteins were commonly identified (with more than one unique peptide) from the cell extracts under all of the experimental conditions tested (Table S1.1). Relative abundance and differential expression profiles were identified following quantile normalization (QN) (30) (Table S1.2). The differentially detected proteins were assessed with the Breitling rank sum (RS) test (31) (Tables S1.3 to S1.5); for proteins significantly differently detected under the different conditions (*P* < 0.05), along with descriptors and annotations, see Tables S1.6 to S1.8. For the GI-EU comparison, 28 proteins were significantly differentially detected (20 up, 8 down) (Fig. 4C); for the EI-EU comparison, 115 proteins were significantly differentially detected (54 up, 61 down) (Fig. 4D). To identify phenomenon-dependent effects and to remove effects due to the specific recombinant protein (eGFP or NusA), differentially abundant proteins common to both the GI-EU and EI-EU comparisons were selected (Fig. 4E). This identified 11 common proteins, 8 up, 2 down, and 1 oppositely detected (Table S1.9). For other comparison analyses of protein abundances that are linked to the specific recombinant proteins, see Tables S1.10 and S1.11.

10.1128/mBio.02118-17.7 FIG S7  PCA, signature, and fraction analysis. (A) PCA analysis of extract fractions of control strain (EU_14-18) and eGFP expression strain (GI_50-54) replicates with outliers (GI_51) highlighted. (B) PCA analysis of extract fractions of control strain (EU_14-18) and eGFP expression strain (GI_50, 52 to 54) replicates with outliers (GI_51) removed. (C) Upregulated proteome level analysis of previously reported downregulated signatures S1 to S32 in wild-type and/or knockout *E. coli* strains (see Table S3) with an expanded view of proteomes with significant enrichment for signatures following mupirocin treatment (S16) (37) and amino starvation (S20) (49). (D) Western blot analysis, performed with anti-*mscL* antibody, of membrane fractions collected from parental and ArfA-deficient (Δ*arfA*) BL21(DE3) strains and of total cells expressing recombinant His-tagged MscL (pmscL). For panel A, the enrichment analysis was performed by the iGA method. *P* values are corrected for multiple testing by the Bonferroni method (74). In panel B, data are presented as the mean value ± the standard deviation of five biological replicates. Download FIG S7, PDF file, 0.2 MB. Copyright © 2018 Morra et al. 2018 Morra et al. This content is distributed under the terms of the Creative Commons Attribution 4.0 International license.

10.1128/mBio.02118-17.8 TABLE S1  Cell extract proteomics data and analysis. Download TABLE S1, XLSX file, 1 MB. Copyright © 2018 Morra et al. 2018 Morra et al. This content is distributed under the terms of the Creative Commons Attribution 4.0 International license.

### GO and subcellular topology enrichment analysis of cell extract.

**FIG 5:**
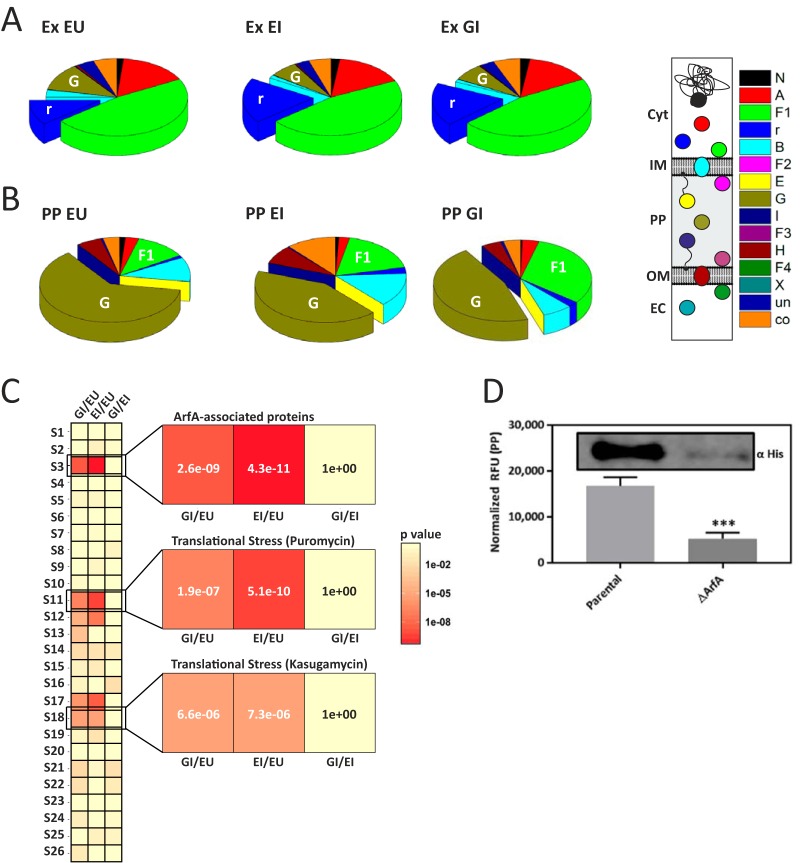
Relative protein abundance with subcellular topological annotation and signature response heat map analysis. (A and B) Relative protein abundance for extract (A) and periplasm (B) clustered by subcellular topology in BL21(DE3)pET-NusA in the absence (EU) or presence (EI) of IPTG and in BL21(DE3)pET-eGFP in the presence of IPTG (GI). N, nucleoid associated; r, ribosomal; A, cytoplasmic; F1, peripherally associated with the IM facing the cytoplasm; B, integral IM proteins; F2, peripherally associated with the IM facing the periplasm; E, IM lipoproteins; G, periplasmic; I, OM lipoproteins; F3, peripherally associated with the OM facing the periplasm; H, integral OM proteins; F4, peripherally associated with the OM facing the extracellular space; X, extracellular space; un, unassigned; co, coannotated. (C) Enrichment analysis of upregulated proteome against stress response signatures S1 to S26 (Table S3) with expanded views of ArfA-associated proteins (S3) (36) and puromycin (S11) and kasugamycin (S18) treatments (37). (D) Periplasmic localization of eGFP in parental and ArfA-deficient (Δ*arfA*) BL21(DE3) confirmed by Western analysis (inset, representative of three biological replicates). Panels A and B, subcellular topology annotation (STEPdb) (34). Panel C, enrichment analysis performed by the iGA method. *P* values were corrected for multiple testing by the Bonferroni method. In panel D, the data are the mean value ± the standard deviation of at least three biological replicates. *, *P* < 0.05; ***, *P* < 0.001; ****, *P* < 0.0001 (two-way ANOVA followed by Dunnett’s multiple-comparison test).

Enrichment analysis was performed by the iterative group analysis (iGA) method (32) after the annotation of detected proteins with gene ontology (GO) terms (AmiGO 2) (33). Upregulated proteins in the GI-EU comparison displayed enrichment for proteins annotated with ribosomal ontology terms (GO.0003735, *P* = 2.5 × 10^−10^; GO.0019843, *P* = 4.1 × 10^−9^) (Table S1.12). Similarly, upregulated proteins in the EI-EU comparison displayed enrichment for proteins assigned to ribosomal ontology terms (GO.0003735, *P* = 1.3 × 10^−15^; GO.0006412, *P* = 4.9 × 10^−11^; GO.0019843, *P* = 6.5 × 10^−11^) and downregulated proteins displayed enrichment for proteins of the OM-bounded periplasmic space (GO.0030288, *P* = 3.2 × 10^−7^). The changes in protein abundance were further explored by clustering the detected proteins into subcellular topological clusters (34), and the relative total abundance for each topology cluster was plotted (Fig. 5A). Upon induction (EI or GI), an increase in protein abundance associated with ribosomal proteins (r) was observed, in addition to a decrease in periplasmic proteins (G). These qualitative observations are further supported by iGA analysis of topological enrichment (Table S1.13). Upregulated proteins in the GI-EU comparison displayed enrichment for genes assigned with a ribosomal topology (r, *P* = 5.7 × 10^−11^), and downregulated proteins displayed enrichment for a periplasmic topology (G, *P* = 2.9 × 10^−6^). Similarly, upregulated proteins in the EI-EU comparison displayed enrichment for proteins assigned with ribosomal topology (r, *P* = 1.5 × 10^−17^) and downregulated proteins displayed enrichment for a periplasmic topology (G, *P* = 1.6 × 10^−13^).

### Protein identification in the periplasmic fraction.

In total, 412 proteins were commonly identified in the periplasmic fraction under all of the experimental conditions tested (Table S2.1). For the significantly differently detected proteins (*P* < 0.05), along with descriptors and annotations, see Tables S2.6 to S2.8. In the GI-EU comparison, 15 proteins were significantly differentially detected (8 up, 7 down), and in the EI-EU comparison, 20 proteins were significantly differentially detected (14 up, 6 down). As in the cell extract analysis, differentially abundant proteins common to both the GI-EU and EI-EU comparisons (2 up, 2 down) were selected (Table S2.9). Interestingly, among the cytoplasmic proteins that were found more abundantly in the periplasm, DnaK, GroEL, and elongation factor Tu 1 (EF-Tu) have previously been described as being nonclassical secreted proteins (5).

10.1128/mBio.02118-17.9 TABLE S2  Periplasm proteomics data and analysis. Download TABLE S2, XLSX file, 0.4 MB. Copyright © 2018 Morra et al. 2018 Morra et al. This content is distributed under the terms of the Creative Commons Attribution 4.0 International license.

### GO and subcellular topology enrichment analysis of the periplasmic fraction.

Enrichment analysis was performed as before (Table S2.12). Proteins less abundantly detected in the GI-EU comparison displayed enrichment for proteins of the OM-bounded periplasmic space (GO.0030288, *P* = 7.5 × 10^−13^). The more abundantly detected proteins in the EI-EU comparison displayed enrichment of ribosomal proteins (GO.0003735, *P* = 5.3 × 10^−8^), and less abundantly detected proteins were enriched for proteins of the OM-bounded periplasmic space (GO.0030288, *P* = 8.6 × 10^−7^). The changes in protein abundance/localization were explored further by clustering the detected proteins into topological groupings (34), and the relative total abundance of each topology cluster was plotted (Fig. 5B). An increase in the relative abundance of proteins peripherally associated with the IM facing the cytoplasm (F1) under induced conditions (EU, 12%; EI, 18%; GI, 31%) was observed. In addition, a decrease in the relative abundance of periplasmic protein (G) from the uninduced conditions (EU, 62%) to the induced conditions (EI, 42%; GI, 46%) was observed. Consistent with this qualitative abundance analysis, iGA topology enrichment analysis demonstrated that the less abundantly detected proteins in the GI-EU comparison displayed enrichment for genes with a periplasmic topology (G, *P* = 1.0 × 10^−15^) (Table S2.13). For the EI-EU comparison, more abundantly detected proteins displayed an enrichment of proteins associated with the ribosome (r, *P* = 9.6 × 10^−9^), and less abundantly detected proteins were associated with the periplasmic compartment (G, *P* = 2.0 × 10^−9^).

### Signature analysis of the cell extract indicates that the excretion phenomenon is associated with translation stress response.

To probe protein expression changes further, an extensive number of enrichment analyses were performed against previously identified stress response signatures by using transcriptomic and proteomic databases (35). By iGA enrichment analysis, a number of stress responses associated with the observed changes in protein level were identified (Fig. 5C; Fig. S7C and Table S3). The most significant enrichment (S3, *P* < 2.6 × 10^−9^) was observed between proteins upregulated under induced conditions in our proteomics data and proteins detected following pulldown with ArfA (Table S3) (36). Significant enrichments (S11, *P* < 1.9 × 10^−7^; S18, *P* < 6.6 × 10^−6^) were also observed among proteins upregulated under induced conditions in our proteomics data that were enriched for proteins encoded by transcripts that are upregulated following puromycin and kasugamycin treatment, respectively (Table S3) (37). Both puromycin and kasugamycin are known to target the ribosome and cause translation stress, and intriguingly, puromycin has also been shown to alleviate growth arrest when SsrA is depleted in a Δ*arfA* mutant strain (36).

10.1128/mBio.02118-17.10 TABLE S3  Signature proteomics analysis. Download TABLE S3, XLSX file, 0.1 MB. Copyright © 2018 Morra et al. 2018 Morra et al. This content is distributed under the terms of the Creative Commons Attribution 4.0 International license.

### Excretion is dependent upon ArfA under conditions of enhanced recombinant protein expression.

As recombinant overexpression is greatly enhanced by T7 RNAP (18, 19) and overproduction of recombinant proteins is known to cause translational stress (38, 39), we explored the association between MscL-dependent excretion and ArfA in the following strains: parental BL21(DE3) and ArfA deficient (Δ*arfA* mutant) expressing eGFP (pET-eGFP), and parental K12 and ArfA deficient (Δ*arfA* mutant) expressing eGFP (pBAD-eGFP). The results show a significant decrease in the periplasmic localization of eGFP in the BL21(DE3) Δ*arfA* mutant strain compared to that in the BL21 (DE3) parental strain (3.2-fold, *P* = 5 × 10^−4^) (Fig. 5D), validating the association of *arfA* with the excretion phenomenon. However, no significant difference in periplasmic localization was observed between the K-12 Δ*arfA* mutant strain and the K-12 parental strain (Fig. S6C), indicating that excretion is dependent upon *arfA* only under conditions of enhanced recombinant expression. Finally as the *mscL* and *arfA* genes overlap, Western blot analysis of the membrane fraction was performed to detect the presence of MscL in the BL21(DE3) parental and *arfA*-deficient strains, confirming the association of the excretion phenomenon with the *arfA* gene (Fig. S7D).

### Excretion of cytoplasmic protein is observed under conditions of translational stress in wild-type cells.

**FIG 6:**
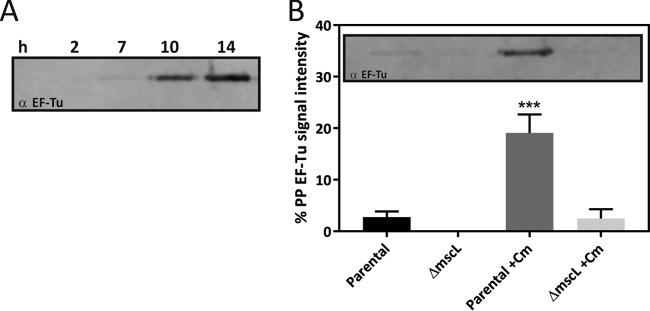
Excretion of cytoplasmic protein EF-Tu in the periplasm following translation stress. (A) Western blot analysis of EF-Tu localization in the periplasmic (PP) fraction from parental *E. coli* K-12 in the presence of Cm (0.01 mg/ml) over time. (B) Periplasmic localization of EF-Tu shown as a percentage of the total (periplasm [PP] plus spheroplast [SP]) protein, in parental and MscL-deficient *E. coli* K-12 cells in the absence or presence of Cm (0.01 mg/ml) after 10 h of treatment. A representative Western blot analysis is shown as an inset. Data in panel B are presented as the mean value ± the standard deviation of three biological replicates. ***, *P* < 0.001 (two-way ANOVA followed by Dunnett’s multiple-comparison test).

To explore whether the observed excretion is an artifact of recombinant protein expression or indeed a native physiological response, we examined if endogenous cytoplasmic proteins are similarly excreted from the cytoplasm of wild-type cells under conditions of translation stress. Cm was used because it is known to cause translation arrest (40) and is reported to cause upregulation of ArfA (41). Medium osmolality was monitored, and Cm was added to cells during the late exponential growth phase, when the medium osmolality had decreased (~Δ70 mOsm). Following Cm addition, an MscL-dependent change in the periplasmic protein profile was detected (Fig. S6D), consistent with the excretion observed in the recombinant system (Fig. S2C). Specifically, Western blot analysis indicates that the cytoplasmic protein EF-Tu accumulates in the periplasm over time upon the addition of Cm (Fig. 6A). In agreement with previous reports (26), basal levels of EF-Tu were detected in the periplasm in an MscL-dependent manner (Fig. 6B). However, upon the addition of Cm to wild-type cells, a large increase in EF-Tu was observed in the periplasm (16-fold, *P* = 5 × 10^−4^), and this excretion was also confirmed to be dependent upon the presence of MscL. Attempts to induce translation stress with Cm in a Δ*arfA* mutant strain led to failure of the cellular fractionation procedure, possibly indicating that under conditions of translation stress, cells exhibit a compromised IM in the absence of ArfA. Taken together, these results indicate that in both recombinant and wild-type cells, the MscL-dependent excretion phenomenon is positively regulated under conditions of translation stress via the action of *arfA*.

## DISCUSSION

Here we report the discovery of a novel signal peptide-independent IM excretion phenomenon in *E. coli* that is triggered by both osmotic stress and translation stress. We have shown that abundant (recombinant) signal peptide-less proteins are released into the extracellular medium in substantial quantities and that this is not associated with a loss of cell viability. However, an increase in the permeability of both the OM and IM was observed in cells exhibiting this phenomenon. Recombinant protein was unexpectedly observed in the periplasmic space >3 h postinduction, prior to being released into the extracellular medium in a manner not associated with OMV encapsulation. As previous studies have shown that during recombinant expression, the OM can become compromised, releasing the periplasmic contents (42, 43), we focused here on the IM phenomenon.

The observed phenomenon of recombinant protein excretion across the IM occurs in an MscL-dependent manner when expression is driven by either a native *E. coli* RNAP or bacteriophage T7 RNAP. MscL is known to protect cells against hypo-osmotic shock, operating via a turgor pressure-dependent gating mechanism, and releases cellular contents to avoid excessive cell expansion (44). The exact pore size of the MscL channel is not known, and no X-ray structures of the open complex have been resolved to date. However, the consensus pore size of ~30 Å, equivalent to a cross-sectional area of ~7 nm^2^ (45), is consistent with the cross-sectional area of the proteins that we report to be excreted across the IM in an MscL-dependent manner (ranging from 27 kDa/~4.5 nm^2^ [eGFP] to 58 kDa/~7 nm^2^ [NusA]) (46). Because of the complexity of the intracellular components, we were unable to measure the osmolality of the cell lysate. However, differential detection of the hyperosmotic shock response osmolyte putrescine suggests that the strains undergoing the excretion phenomenon are experiencing greater hypo-osmotic (less hyperosmotic) conditions. In addition, this notion is consistent with the IM permeability observed in cells undergoing excretion (Fig. 2D) and in agreement with previous studies showing that cells under conditions of hypo-osmotic shock are stained by PI (16). During cell culture, we observed a decrease in medium osmolality of ~100 mOsm. Even on the basis of presuming no change in the internal osmolyte concentration, this would result in an increase in turgor pressure of ~2.5 atm (40 mOsm = ~1 atm), above the gating threshold (~0.2 atm) observed for MscL channels in isolated membranes (44). In contrast to previous studies, we have found that medium exchange is not required to elicit MscL-dependent protein excretion.

GO and subcellular topology analysis of proteins that increase in relative intracellular abundance during the excretion phenomenon displayed a significant enrichment for ribosomal proteins. This is surprising, as recombinant protein expression has previously been shown to lead to depletion of ribosomal proteins (39, 47). Additionally, depletion of amino acid is also known to lead to stringent-response-mediated depletion of ribosomal proteins (48, 49). Proteins that decrease in abundance were found to be significantly enriched with periplasmic proteins, consistent with the loss of the periplasmic contents during the observed phenomenon. Among the cytoplasmic proteins increasing in abundance in the periplasmic fraction, some were previously detected in the extracellular space (8), including DnaK and GroEL (5).

The proteome signature of the excretion phenomenon was found to be significantly associated with proteins that bind to ArfA (*P* < 2.6 × 10^−9^) and with the response to antibiotic treatment targeting the ribosome (puromycin; *P* < 1.9 × 10^−7^). Interestingly, puromycin is believed to act in a manner similar to that of ArfA in that they both enter the A site of the ribosome and cause dissociation of the ribosome complex. Indeed, puromycin and ArfA lead to premature nascent chain release and release of nonstop mRNA from the ribosome, respectively (36, 50). Furthermore, puromycin has been shown to alleviate growth suppression in an SsrA-depleted Δ*arfA* mutant strain (36). To validate the association between *arfA* and the phenomenon, a genetic knockout of *arfA* was created in BL21(DE3), which led to significantly less excretion into the periplasm (Fig. 5D). In contrast, when expression was driven from an *E. coli* RNAP-dependent promoter (P_*BAD*_), no significant reduction of periplasmic excretion was observed in the absence of *arfA* (Fig. S6C). This difference in the dependency upon *arfA* is most likely due to the lower levels of recombinant expression, and hence reduced translation stress, under control of the *E. coli* RNAP-dependent promoter. Therefore, in the absence of *arfA*-mediated translation stress, MscL-dependent excretion is triggered by only osmotic stress. Finally, and with the most relevance for native cell physiology, the phenomenon was also observed in wild-type (nonrecombinant) cells undergoing translational stress (Fig. S6D). Specifically, the cytoplasmic protein EF-Tu was excreted into the periplasm in an MscL-dependent manner (Fig. 6). Previously, basal levels of EF-Tu have been detected in the periplasm following osmotic downshock (26). However, in this study, the basal excretion is greatly enhanced and is positively regulated in response to translational stress in both recombinant and wild-type cells.

The findings presented here suggest that the observed MscL-dependent excretion is a native response to translation stress via the action of *arfA*. Rescue of stalled ribosomes is predominantly mediated by the *trans*-translation system through the action of transfer messenger SsrA (tmRNA) and small protein B (SmpB). The tmRNA-SmpB complex alleviates the stalling and targets the mRNA and nascent protein for degradation (51, 52). When the *trans*-translation system is compromised, alternative ribosome rescue systems A and B (ArfA and ArfB) are used. As the *trans*-translation rescue pathway consists of a degradative function to recycle mRNA and amino acids, whereas ArfA does not, it is tempting to speculate that MscL may possibly play an excretory role in the ArfA-mediated response to translation stress. This would presumably also result in the nonspecific release of cytoplasmic contents. Why cells would utilize such an energetically inefficient rescue mechanism is not entirely clear. Intriguingly, *arfA* is located adjacent to *mscL* in the *E. coli* genome, an arrangement that is also evolutionarily conserved across other bacterial species (53). Small RNAs from the overlapping transcripts of both *arfA* and *mscL* have been detected (54, 55); detailed mechanistic analysis will be required to dissect how posttranscriptional control may regulate MscL-dependent excretion in response to osmotic and translation stress.

## MATERIALS AND METHODS

### Strains and plasmids used in this study.

*E. coli* BL21(DE3) (Novagen) and K-12 BW25113 parent and Keio Knockout Collection strains (28), BL21(DE3) Δ*arfA* and Δ*mscL* mutants (generated in this work), pBAD (Thermo Fisher), pET expression vectors (Novagen), pRL128 (Addgene 40180), and pSIM18 (gift from SynBioChem, University of Manchester) were used in this study. The eGFP-encoding gene bearing a 5′ His tag sequence was cloned into pET44 and pBAD, affording the pET44_eGFP and pBAD_eGFP vectors, respectively, in which no signal peptide or Nus tag was present. The *nusA* gene bearing 5′ and 3′ His tags is present in the pET44 vector and was subcloned into the pBAD vector. The AcDKR-encoding gene (pET26b-AcDKR vector) was a kind gift from Reddy’s Laboratories (EU) Ltd. The pectate lyase gene (pET24a-PL) was a kind gift from Biocatalysts Ltd. The scFv13R4-encoding gene was synthesized (GeneArt) and subcloned into both pET44 and pBAD vectors.

### Expression induction procedure.

All cultures were grown in TB (2.7% yeast extract, 4.5% glycerol, 1.3% Bacto tryptone) or, where specified, in LB (10 g/liter NaCl, 5 g/liter yeast extract, 10 g/liter tryptone) supplemented with 0.2% glucose and inoculated directly from freshly plated single colonies. For exponential/transition growth phase experiments (0 to 12 h postinduction), precultures were grown at 20°C with shaking (180 rpm) to an optical density at 600 nm (OD_600_) of ~0.8 and then transferred to Ultra Yield (UY) flasks (Thomson) with or without an appropriate inducer (I or UI). Cultures were then grown at 30°C with shaking (210 rpm) for up to 12 h. For late exponential/transition growth phase experiments (12 to 24 h postinduction), precultures were grown at 37°C with shaking (180 rpm) to an OD_600_ of ~0.8 and then transferred to UY flasks with or without an appropriate inducer (I or UI). Cultures were then grown at 30°C with shaking (210 rpm) for up to 24 h. For the nonrecombinant system experiments, preinoculums were diluted to an OD_600_ of ~0.01, transferred to UY flasks, and grown at 30°C with shaking (210 rpm) for 14 h (OD_600_ of ~20). Cm (0.01 mg/ml, final concentration) was added, and cells were assessed at the time points indicated. The inducers 250 μM IPTG (Sigma), 13.3 mM arabinose (Sigma), and 1 mM rhamnose (Sigma) were used as required. The antibiotics ampicillin (100 µg/ml), kanamycin (50 µg/ml), and Cm (100 µg/ml) were used as required. A BMG ClarioStar microplate reader was used to measure the colorimetric fluorescence and cell density (OD_600_) of intact cells.

### Expression analysis and quantification.

To measure extracellular eGFP expression, cell cultures were harvested by centrifugation (4,500 × *g*) and the relative florescence units (RFU) were assessed directly. To measure intracellular eGFP expression, harvested cells were washed twice in equal volumes of phosphate-buffered saline (PBS) with 0.2% Tween. RFU and OD_600_ were measured, and normalized (RFU/OD) values were plotted against the time postinduction. For SDS-PAGE and Western blot analyses, OD-normalized volumes were collected and resuspended in SDS-PAGE loading buffer. Equal volumes were loaded onto a 4 to 20% gradient gel (Bio-Rad) and separated by SDS-PAGE, and target proteins were confirmed by Western blot analysis. Membranes were blocked with 5% skimmed milk in PBS containing 0.2% Tween. His-tagged recombinant proteins (eGFP, scFv, NusA) were detected with a mouse anti-His monoclonal antibody (Thermo Fisher MA1-21315, 1:3,000 in 5% skimmed milk) and a IRDye anti-mouse IgG secondary antibody (LI-COR, 1:10,000 in 5% skimmed milk). Endogenous MscL protein was detected on total membrane (collected by ultracentrifugation from sonicated cells after growth in LB supplemented with 0.5 M NaCl) with an anti-MscL antibody (kindly provided by P. Blount, University of Texas Southwestern Medical Center). When required, anti-RNAP 70 (Abcam, Inc., ab12088, 1:3,000 in 5% skimmed milk) and anti-beta lactamase (Novus Biologicals, 8A5-A10, 10 µg/ml in 5% skimmed milk) were used as fractionation control. The infrared fluorescence of IRDye secondary antibodies was detected with an Odyssey imager (LI-COR). SDS-PAGE gels stained with Instant Blue (Expedeon) or Sypro red (Thermo Fisher) protein gel stain were assessed by densitometry analysis with ImageJ or Image Studio (LI-COR) software. Protein quantification was performed with purified recombinant protein and/or bovine serum albumin (BSA) standard curves. All data were measured in biological triplicates.

### scFv β-Gal binding assay.

Two-microliter β-Gal (0.3 mg/ml) dots were spotted directly onto a nitrocellulose membrane (Amersham Hybond). As a negative control, the same amounts of BSA were spotted onto the same membrane. The membrane was left to dry for 5 min. Nonspecific binding sites were blocked by incubating the membrane with 5% milk in PBS for 1 h (50 rpm, room temperature [RT]). The membrane was washed three times for 15 min with PBS and then left to dry. On each dot, the substrate was added as 2 μl of a serial dilution of the periplasmic fraction containing scFv13R4 (previously quantitated by Western blot analysis) and allowed to dry for 5 min. The membrane was blocked with 5% milk in PBS for 20 min (50 rpm, RT). His-tagged scFv13R4 protein was detected as described above for expression analysis and quantification. The signal intensity was quantified with the Image Studio 5.0 software for densitometry analysis and used for curve fitting by using a four-parameter logistic function. All data were measured in biological triplicates.

### AcDKR activity assay.

The extracellular fraction of a BL21(DE3)pET-AcDKR culture was used to assay the activity of the recombinant protein by a UV kinetic activity assay measuring the absorbance change at 340 nm because of the oxidation of NADH over a 5-min period. Ethyl 4-chloroacetoacetate (Sigma-Aldrich 180769) was used as the substrate. The activity of the enzyme was calculated in units per milliliter (micromoles per milliliter per minute) of NADH consumed. All data were measured in biological duplicates.

### CFU assay.

Cultures were grown as described above for the expression induction procedure. Ten-microliter drops of serially diluted (1:10) cultures were spotted onto agar plates containing the appropriate antibiotic. Colony formation was observed after incubation overnight at 37°C. The colonies were counted and normalized for an OD_600_ of 1.0 to calculate the normalized number of CFU per milliliter. All data were measured in biological triplicates.

### OMV purification.

OMVs were isolated as previously described (22). In brief, the following steps were performed. (i) The extracellular fraction was collected from UY cultures by low-speed centrifugation (4,500 × *g* for 15 min), which removed most of the bacteria, the rest of which were eliminated by sterile filtration through a 0.45-µm filter. (ii) Centrifugation at high speed (35,000 × *g*) of the filtered extracellular fraction was followed by ultracentrifugation (200,000 × *g*). (iii) The pellets (OMVs) were resuspended in 1 ml of 20 mM Tris (pH 8). Target proteins were confirmed by Western blot analysis with anti-OmpA (Antibody Research 111120, 1:20,000 in 5% skimmed milk), anti-GroEL (Sigma G6532, 1:20,000 in 5% skimmed milk), and anti-His (Thermo Fisher MA1-21315, 1:3,000 in 5% skimmed milk) antibodies. OMVs were prepared from biological triplicates.

### IM permeability assays.

*E. coli* BL21(DE3)pET44-eGFP was grown as described above for the expression induction procedure. At the postinduction time points indicated, ~10^9^ cells (OD_600_ of 1) were collected, washed, and resuspended in 1 ml of PBS (~10^9^ cells/ml). Ten microliters (~10^7^ cells) were added to 96-well microtiter plates containing 90 µl of 0.4 mg/ml *o*-nitrophenyl-β-d-galactopyranoside (ONPG) substrate. The assay mixture was incubated at 30°C for 90 min (the reaction was stopped by adding 5 mM Na_2_CO_3_), and *o*-nitrophenol generation was monitored at 420 nm. Cells permeabilized with chloroform (100 µl) and 0.1% SDS (50 µl) and incubated for 5 min at 28°C were used as a positive control. The data are expressed in blanked corrected absorbance units. All data were measured in biological triplicates. For fluorescence-activated cell sorting (FACS), cells were washed once and then diluted 1/1,000 in PBS. The fluorescent dye PI was added to the cells at a concentration of 7.4 µM. Flow cytometry was performed with a Sony SH800 Cell Sorter (Sony Biotechnology) equipped with a 488-nm laser, enabling excitation of eGFP and PI, with subsequent emission being measured through use of the 525- and 617-nm channels, respectively. Data were recorded for 100,000 cells per sample at ≤5,000 events/s, fluorescence compensation was applied, and analysis was performed with SH800 software. Histograms of events versus eGFP and PI fluorescence were plotted and used to determine the mean fluorescence and coefficients of variation of the total population and gated subpopulations consisting of nonfluorescent and fluorescent cells. Additionally, the percentage of total events falling within each gated subpopulation was recorded. All data were measured in biological duplicates.

### OM permeability assays.

*E. coli* BL21(DE3)pET26b-AcDKR was grown as described above for the expression induction procedure. At the postinduction time points indicated, ~10^9^ cells (OD_600_ of 1) were collected, washed, and resuspended in 1 ml of PBS (~10^9^ cells/ml). Ten microliters (~10^7^ cells) was added to 96-well microliter plates containing 90 µl of 20 µM NPN in PBS. The change in fluorescence at 420 nm following 30 min of incubation at 30°C was recorded. Permeabilized cells (OD_600_ of ~1) with the addition of 1 mM of EDTA and incubation at RT for 3 min were used as a positive control. The data are expressed in blanked corrected fluorescence units. All data were measured in biological duplicates.

### Fractionation of *E. coli*.

Cultures were grown as described above for the expression induction procedure. At the postinduction time points indicated, ~10^9^ cells (OD_600_ of 1) were collected, washed in PBS, and resuspended in 250 μl of buffer I (100 mM Tris-acetate [pH 8.2], 500 mM sucrose, 5 mM EDTA). A 2.7-µl volume of 15 mg/ml lysozyme (Sigma) was added, immediately followed by 250 μl of pure water (Milli-Q). The samples were incubated on ice for 5 min, after which 10 µl of 1 M MgSO_4_ was added. The spheroplast (pellet) and periplasm (supernatant) fractions were collected by centrifugation at 17,000 × *g* for 10 min at 4°C. The periplasmic fraction was centrifuged again to remove any remaining spheroplasts. The spheroplast fraction was washed twice in buffer II (50 mM Tris-acetate [pH 8.2], 250 mM sucrose, 2.5 mM EDTA, 5 mM MgSO_4_). All fractions were prepared from biological triplicates.

### Knockout of ArfA and MscL in BL21(DE3).

The knockout strains were generated as described previously for Lambda Red recombineering (56), with the pRL128 (template) (57) and pSIM18 (recombination) (58) plasmids. The oligonucleotides used to amplify FRT-flanked kanamycin selection cassettes were H1_*arfA* (ACTGGCAGGATTACACTCGCGCCGTTAAATAACCAACTGGAGTTTTTATGTTGGGATCCGTCGACCTGCA), H2_*arfA* (GATTTGCTGAAAGAGCAGAATAACCGCTCTTAACAAGCGCCTGAAAGCAGGAGGTGTAGGCTGGAGCT), H1_*mscL* (TTAACATTTGTTAGACTTATGGTTGTCGGCTTCATAGGGAGAATAACATGTTGGGATCCGTCGACCTGCA), and H2_mscL (ACCACTGGTCTTCTGCTTTCAGGCGCTTGTTAAGAGCGGTTATTCTGCTCGAGGTGTAGGCTGGAGCTGC).

### Sample preparation for GC-MS.

Samples for metabolic analyses (in five biological replicates) were taken from BL21(DE3) shake flask cultivations carrying either pET44_eGFP or pET44_NusA and grown for 16 h as described above for the expression induction procedure. Samples were collected for metabolic profile analysis by quenching 2-ml aliquots with a chilled 60% aqueous methanol solution (−48°C), followed by 10 min of centrifugation at 6,000 × *g* at −9°C to harvest the biomass. Internal metabolite extraction was carried out with an 80% aqueous methanol solution (−48°C) and a freeze-thaw method as described previously (59, 60). A two-step derivatization protocol was employed for all samples, starting with oximation (with methoxyamine-hydrochloride in pyridine), followed by a silylation step with *N*-methyl-*N*-(trimethylsilyl) trifluoroacetamide (61, 62). For the footprint analysis, samples were collected by centrifuging 1 ml of medium at 6,000 × *g* for 10 min to remove the cellular biomass. The supernatant was collected, quenched in liquid nitrogen, and stored at −80°C. Upon analysis, 200-µl aliquots of samples were transferred to new microcentrifuge tubes; this was followed by combining 100 µl from each of the samples to generate quality control (QC) samples (61, 63). A 100-µl volume of an internal standard solution (0.2 mg/ml each succinic-*d*_4_ acid, glycine-*d*_5_, and lysine-*d*_4_) was added to all samples before they were dried overnight with a speed vacuum concentrator (Concentrator 5301; Eppendorf, Cambridge, United Kingdom).

### GC-MS instrument setup and data processing.

An Agilent 6890N GC oven (Agilent Technologies UK, Wokingham, United Kingdom) coupled to a Leco Pegasus III mass spectrometer (Leco, St. Joseph, MI) was used in conjunction with a Gerstel MPS-2 autosampler (Gerstel, Baltimore, MD) as previously described (63, 64). All collected data were deconvoluted with Leco ChromaTOF software, and the metabolites detected were identified in accordance with the Metabolomics Standards Initiative guidelines (65). The chromatographic peak corresponding to IPTG was removed from the data before any further analysis to avoid any variation resulting from the presence of this compound. By using the QC samples, mass spectral features with high deviation and missing values were removed, followed by normalization of the peak areas according to the internal standard and OD_600_ of each sample. All data collected were analyzed with Matlab version 8 (MathWorks Inc., Natick, MA). All preprocessed GC-MS peak areas were initially subjected to PCA (66), followed by discriminant function analysis (PC-DFA) where required (67). PCA and PC-DFA loading plots were used to determine the main metabolites contributing to the clustering patterns. The statistical significance (*P* < 0.05) of these metabolites was further confirmed by one-way analysis of variance (ANOVA) and Tukey’s *post hoc* test.

### Sample preparation for proteomics.

Samples for proteomic analyses (in five biological replicates) were taken from BL21(DE3) shake flask cultivations carrying either pET44-eGFP or pET44-NusA and grown for 16 h as described above for the expression induction procedure. Total cell extract (Ex) and periplasmic (P) samples were prepared as follows. (i) Ex samples were prepared from cells harvested at 4,500 × *g*, washed in PBS (twice), resuspended in buffer 1 (10 mM Tris [pH 8] and 200 mM NaCl containing protease inhibitor cocktail and DNase), disrupted by sonication at 30 A for 10 min (20-s on, 40-s off cycle), and centrifuged at 35,000 × *g* for 30 min. (ii) P samples were prepared as described above for the fractionation of *E. coli*. The samples were then incubated with GFP-Trap_A beads (ChromoTek) in accordance with the manufacturer’s protocol to deplete the dominant eGFP. SDS at 0.1% was added to the unbound fraction, which was snap-frozen in liquid nitrogen and stored at −80°C until further processing.

### LC-MS/MS analysis.

Samples were prepared for MS by a modified FASP method (68) with the following modification. Samples were buffer exchanged with 0.1% SDS in Microcon 30-kDa centrifugal filter units (Merck Millipore) at 14,000 × *g*. After three cycles of buffer exchange with 0.1% SDS (50 µl), the protein concentration was measured with a Millipore Direct Detect spectrometer at AM3. A total of 12.5 µg of protein was added to a fresh 30-kDa filter tube for reduction (dithiothreitol), alkylation (iodoacetamide), and proteolytic digestion to be performed in the filter tubes. The SDS buffer in the samples was exchanged with 8 M urea–0.1 M Tris-HCl buffer (pH 8) for two cycles. Following reduction and alkylation (68), the proteins were concentrated and resuspended in 6 M urea in Tris-HCl buffer (pH 8). Endoproteinase Lys-C was used for the initial digestion at a 1:30 enzyme-to-protein ratio; after 3 h, the urea concentration was dropped to 1 M with Tris-HCl (pH 8). The proteins were then digested with trypsin at a 1:50 enzyme-to-protein ratio. After digestion, peptides were collected by centrifugation and desalted with OLIGO R3 reversed-phase resin on a microplate system and then reconstituted in 5% acetonitrile and 0.1% formic acid. Digested samples were analyzed by liquid chromatography-tandem MS (LC-MS/MS) with an UltiMate 3000 rapid separation liquid chromatograph (RSLC; Dionex Corporation, Sunnyvale, CA) coupled to a Q Exactive HF Hybrid Quadrupole-Orbitrap (Thermo Fisher Scientific, Waltham, MA) mass spectrometer. Peptide mixtures were separated by using a 60-min gradient of 92% A (0.1% FA in water) and 8% B (0.1% FA in acetonitrile) to 33% B in 44 min at 300 nl/min with a BEH C18 analytic column (130 Å pore size, 1.7 μm, 75 µm × 250 mm; Waters). Peptides were selected for fragmentation automatically by data-dependent analysis.

### Protein identification and label-free quantification.

The acquired raw files were imported into the Progenesis LC-MS software (v2.0; Nonlinear Dynamics Limited), which was used to extract peptide precursor ion intensities across all samples by applying the default parameters. The proteins were identified by using MASCOT against the predicted proteomes of both *E. coli* K-12 (UniProt version July 2016; narrowed to *E. coli* strain K-12) and *E. coli* BL21(DE3) (UniProt version July 2016; narrowed to *E. coli* strain B/BL21-DE3). The K-12 database consists of 4,303 *E. coli* proteins. The BL21(DE3) database consists of 4,159 *E. coli* proteins. The search criteria were set as follows: fixed modifications, carbamidomethyl (C); variable modifications, oxidation (M); peptide tolerance, 5 ppm; MS/MS tolerance, 0.5 Da; peptide charges, 2+ and 3+. Peptide identification was performed against both the *E. coli* K-12 proteome and the BL21(DE3) proteome (experimental). Since only a small number of additional proteins were identified, all subsequent analysis was performed with the K-12 reference proteome because of its superior annotation.

### Statistical analysis for detection of differentially expressed proteins.

Before statistical analysis was performed, QN was applied to the proteomics data sets (69). QN creates equal distribution across all samples by replacing each data point with the mean of the corresponding quantile. It was performed through the R/Bioconductor package preprocessCore (70). PCA of all 15 samples (three conditions, five replicates) was performed to identify condition clustering; one outlier (GI-51) was removed from the data set and subsequent analysis. The presence of differentially expressed proteins was investigated for all possible comparisons (GI-EUI, EI-EUI, and GI-EI) by RS analysis (31) performed with the R/Bioconductor package RankProd (71, 72). The *P* values obtained were then corrected for multiple testing by the Benjamini-Hochberg method (73).

### iGA.

The iGA method (32) was used to detect overrepresented (enriched) groups of features for each experimental comparison (GI-EU, EI, EU, or GI-EI). The *P* values obtained from the iGA were corrected for multiple testing by the Bonferroni method (74). The protein annotation reported in the STEPdb database (34) was used to assign a specific subcellular topology (location) to each protein. iGA was applied to the list of detected proteins ordered according to the one-tailed *P* values obtained from the RS analysis. In accordance with the topology analysis, enrichment analysis of GO terms in the upregulated (or downregulated) proteins in each comparison was performed by considering all of the GO terms reported in the AmiGO database (33) with two or more members on the list of detected proteins. iGA analysis was used to assess if the detected proteome level changes constitute a unique signature or if they are related to previously studied processes. We collected previously identified transcript/protein signatures for a number of different studies of *E. coli* (wild type and/or knockout strains) (Table S3).

### Accession number(s).

The full data from this study were submitted to ProteomeXchange under project accession no. PXD006474 (project doi:10.6019/PXD006474).
