# Supplementary figures and images for: Translation Stress Positively Regulates MscL-Dependent Excretion of Cytoplasmic Proteins
Source: mBio. 2018 Jan 30;9(1):e02118-17. doi: 10.1128/mBio.02118-17 (PMC5790912; doi:10.1128/mBio.02118-17)

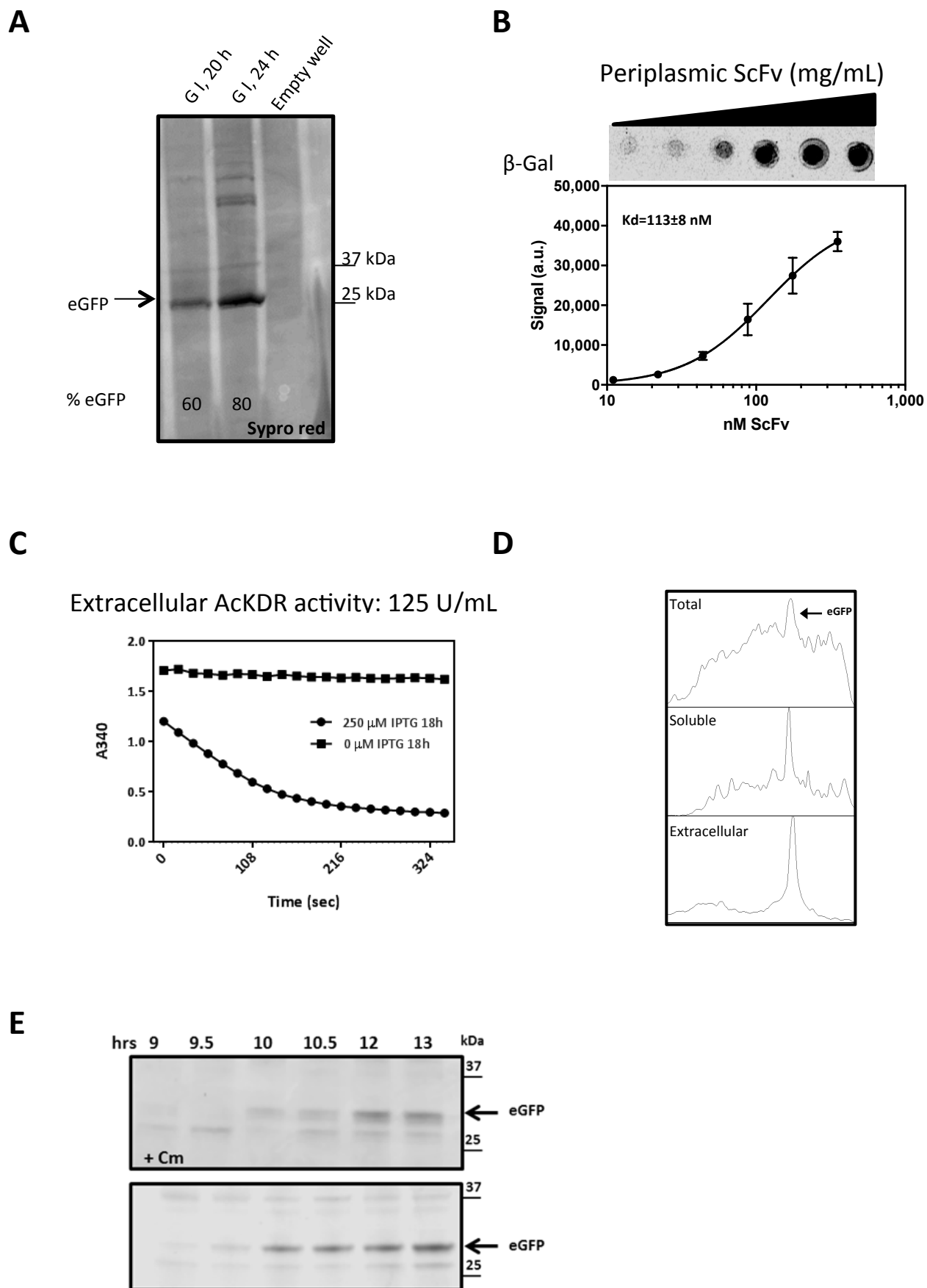

Figure S1

Supplement: FIG S1 [file mbo001183699sf1.pdf]

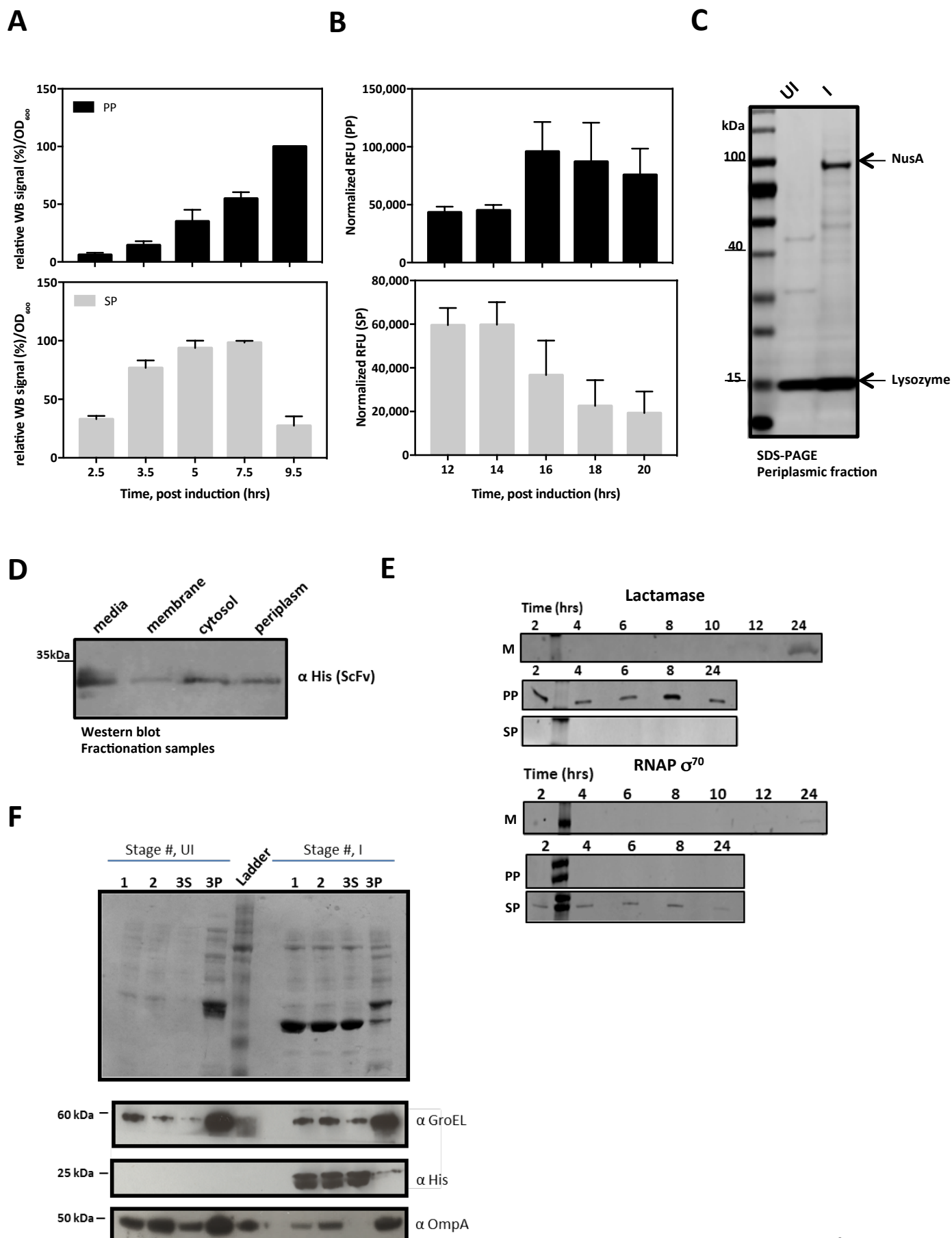

Figure S2

Supplement: FIG S2 [file mbo001183699sf2.pdf]

**A**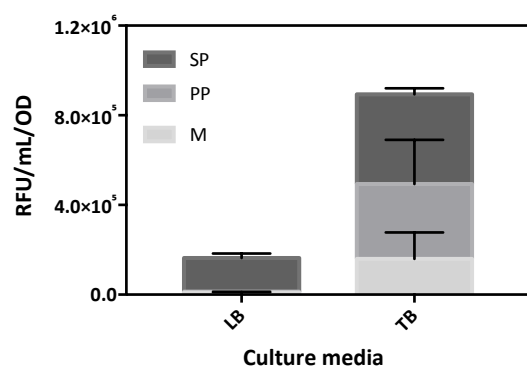**B**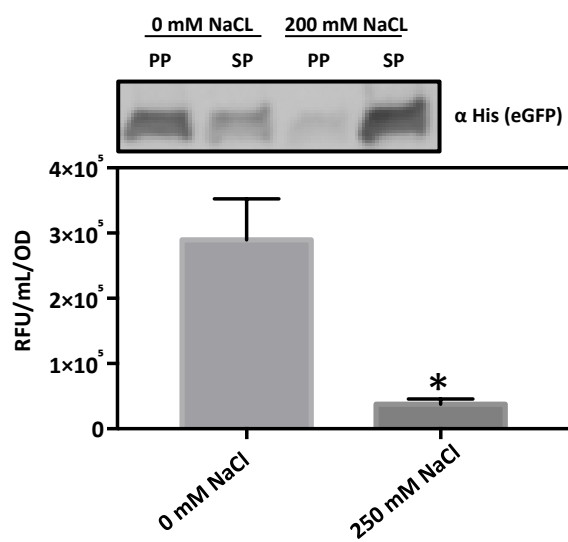**C**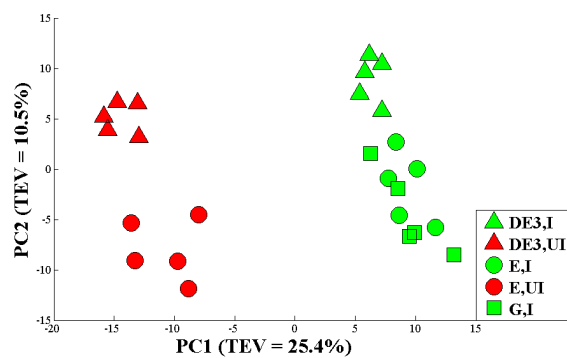**D**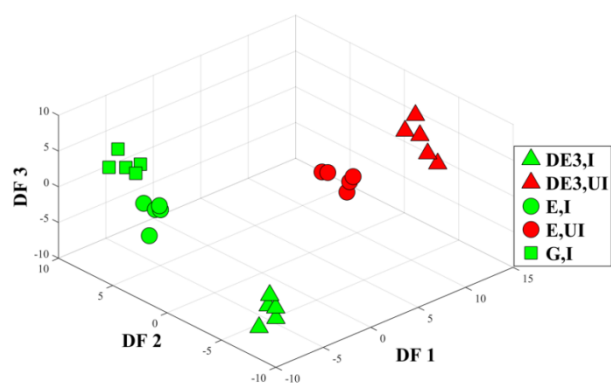**Figure S3**

Supplement: FIG S3 [file mbo001183699sf3.pdf]

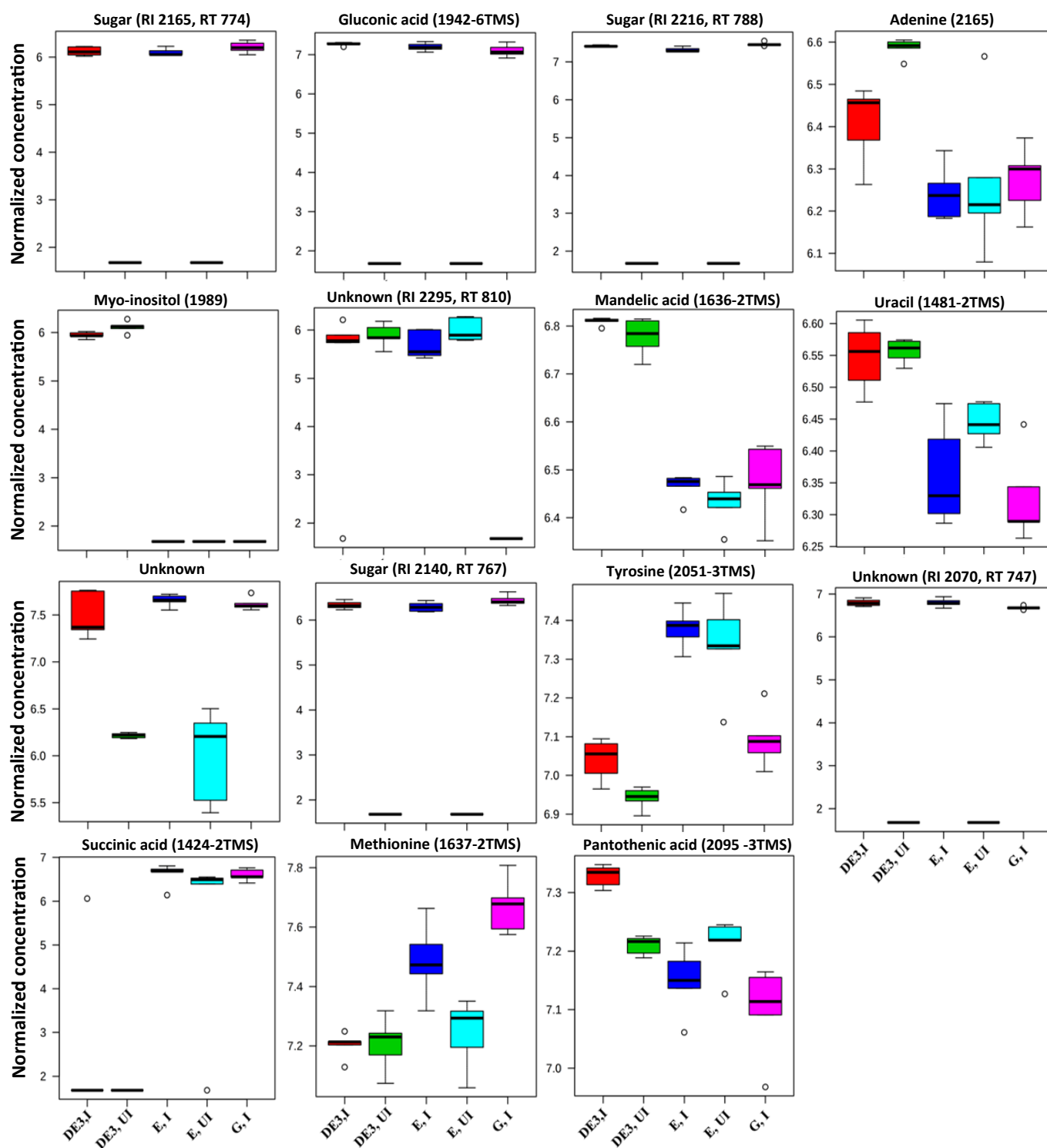

Figure S4

Supplement: FIG S4 [file mbo001183699sf4.pdf]

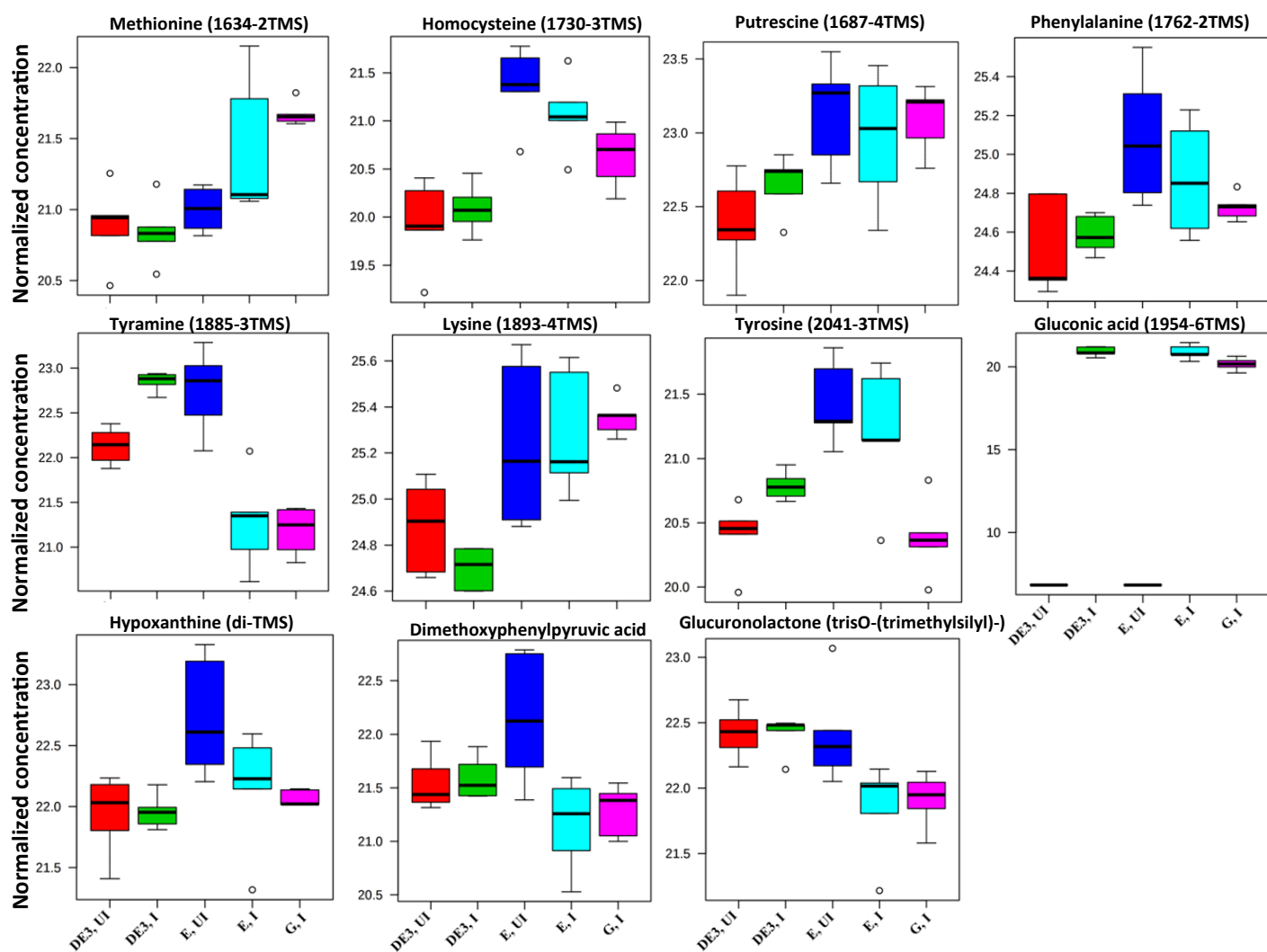

Figure S5

Supplement: FIG S5 [file mbo001183699sf5.pdf]

**A**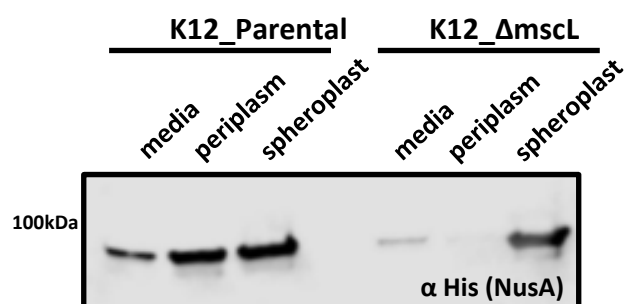**B**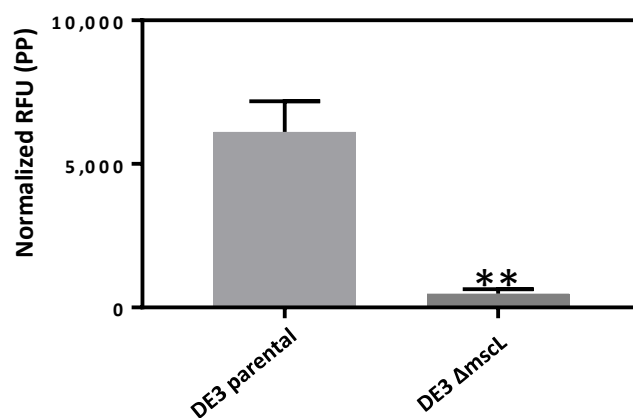**C**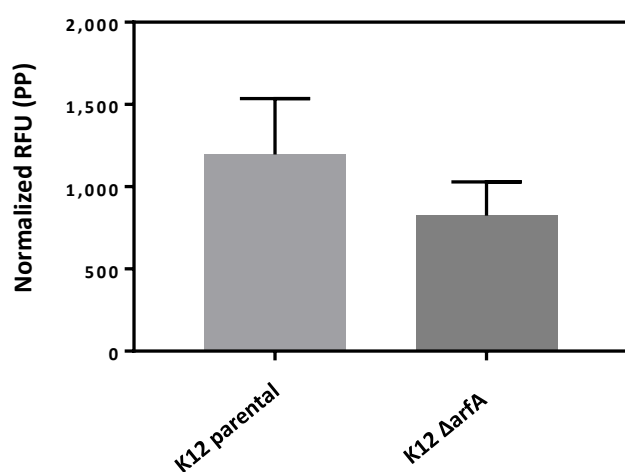**D**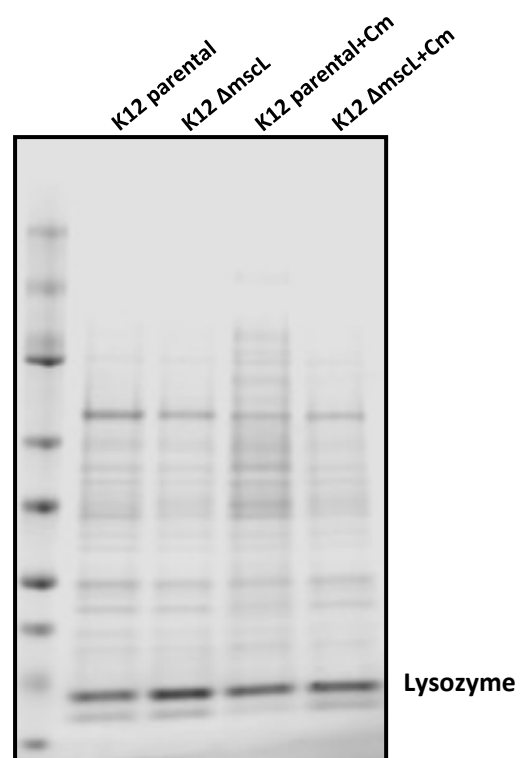**Figure S6**

Supplement: FIG S6 [file mbo001183699sf6.pdf]

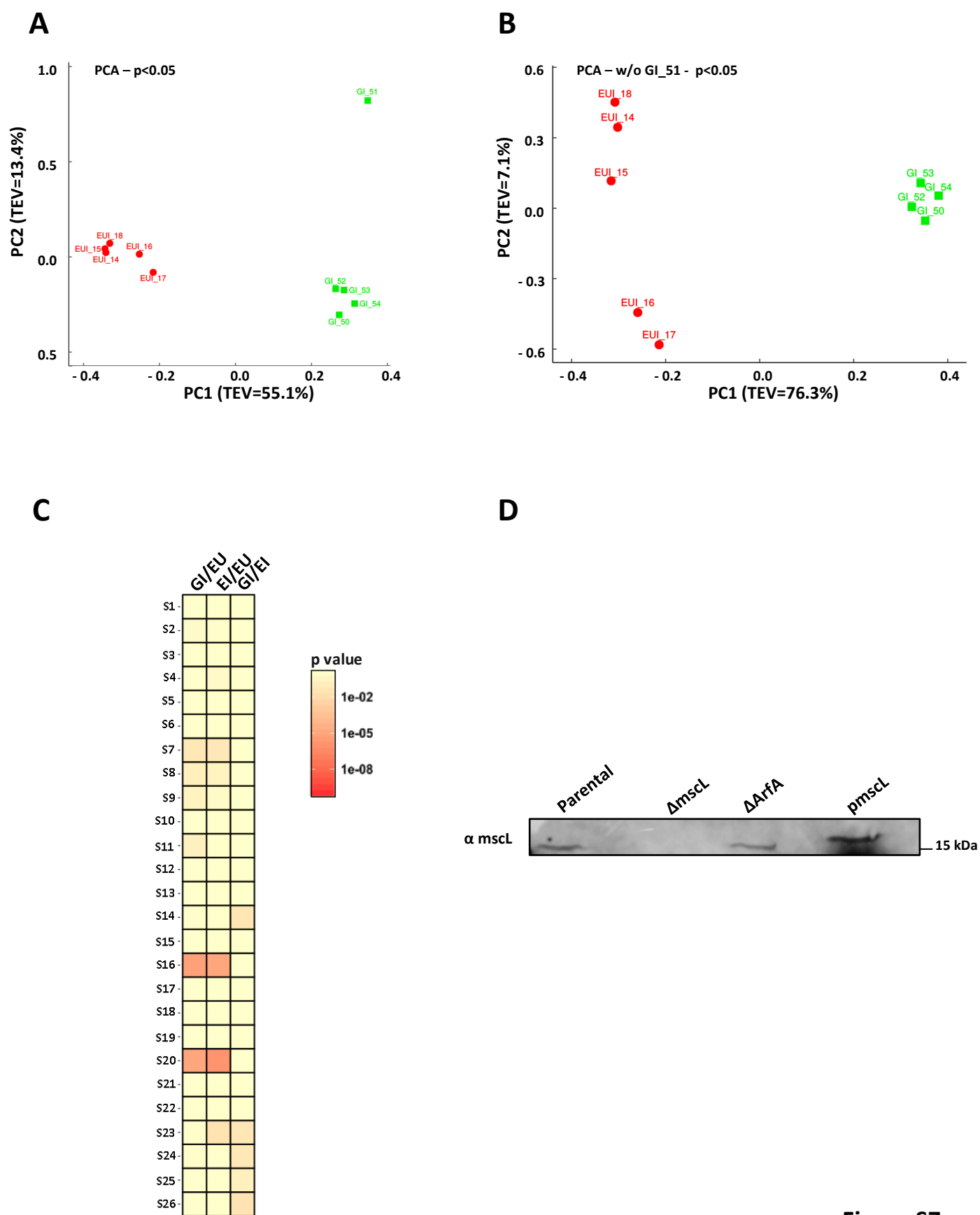

Figure S7

Supplement: FIG S7 [file mbo001183699sf7.pdf]
